# The MYC-regulated lncRNA LNROP (ENSG00000254887) enables MYC-driven cell proliferation by controlling the expression of OCT2

**DOI:** 10.1038/s41419-023-05683-6

**Published:** 2023-02-27

**Authors:** Daniel García-Caballero, Jonathan R. Hart, Peter K. Vogt

**Affiliations:** Scripps Research, Department of Molecular Medicine, 10550 North Torrey Pines Road, La Jolla, CA 92037 USA

**Keywords:** Long non-coding RNAs, Oncogenes, Cancer epigenetics

## Abstract

MYC controls most of the non-coding genome. Several long noncoding transcripts were originally identified in the human B cell line P496-3 and then shown to be required for MYC-driven proliferation of Burkitt lymphoma-derived RAMOS cells. In this study, we used RAMOS cells exclusively as a representative of the human B cell lineage. One of the MYC-controlled lncRNAs required for RAMOS cell proliferation is ENSG00000254887 which we will term LNROP (long non-coding regulator of POU2F2). In the genome, LNROP is located in close proximity of POU2F2, the gene encoding OCT2. OCT2 is a transcription factor with important roles in sustaining the proliferation of human B cells. Here we show that LNROP is a nuclear RNA and a direct target of MYC. Downregulation of LNROP attenuates the expression of OCT2. This effect of LNROP on the expression of OCT2 is unidirectional as downregulation of OCT2 does not alter the expression of LNROP. Our data suggest that LNROP is a cis-acting regulator of OCT2. To illustrate the downstream reach of LNROP, we chose a prominent target of OCT2, the tyrosine phosphatase SHP-1. Downregulation of OCT2 elevates the expression of SHP-1. Our data suggest the following path of interactions: LNROP enables the proliferation of B cells by positively and unidirectionally regulating the growth-stimulatory transcription factor OCT2. In actively proliferating B cells, OCT2 attenuates the expression and anti-proliferative activity of SHP-1.

## Introduction

MYC is a basic helix-loop-helix leucine zipper (bHLHLZ) protein that plays important roles in several cellular programs including proliferation, differentiation, metabolism, apoptosis and maintenance of pluripotency. It is part of a transcription factor network and functions as dimer with the bHLHLZ protein MAX [1–4]. The MYC-MAX dimer binds to a DNA enhancer box element with the consensus sequence CACGTG (E-box) to elevate or suppress gene expression [5]. MYC shows gain of function in numerous human cancers [6, 7]. The classic example of a MYC-driven tumor is Burkitt lymphoma in which a chromosomal translocation brings the expression of MYC under the control of the immunoglobin heavy chain locus [8].

Long noncoding RNAs (lncRNAs) are transcripts with a sequence larger than 200 base pairs, lacking the potential to be translated into proteins [9–13]. They represent up to 70% of the human transcriptome [14]. LncRNAs function in epigenetic regulation by interacting with chromatin components;[15, 16] they serve as guides to regulate gene expression pre- and post-transcriptionally [10, 17]; they can also work as scaffolds for RNA, DNA and protein complexes [18]. The connection between MYC, lncRNAs and cancer has been analyzed in several reviews [19–25].

MYC differs from other transcription factors, because it acts not only on a set of specific downstream target genes but is a general amplifier of transcription bound to most active promoters [26–29]. Using the human B cell line P493-6 that carries a Tet-regulatable MYC transgene, our group and others have shown that MYC controls most of the non-coding transcriptome [30, 31]. These findings are supported by ChIP-seq data showing that MYC-regulated lncRNAs are occupied by MYC at their transcription start site (TSS). Building on these results, we developed a new CRISPRi approach and identified MYC-regulated lncRNAs that are required for cell proliferation in human B cells [32]. Several of the top candidates of this CRISPRi screen are known for their involvement in cancer including DANCR [33–35], MIR17HG [36], SNHG17 [37] and SNHG26 [38, 39]. Here, we have analyzed the function of the transcript ENSG00000254887, referred to as LNROP (long non-coding regulator of POU2F2), which was also identified in the previous CRISPRi screen as indispensable for B cell proliferation.

**Fig. 1:**
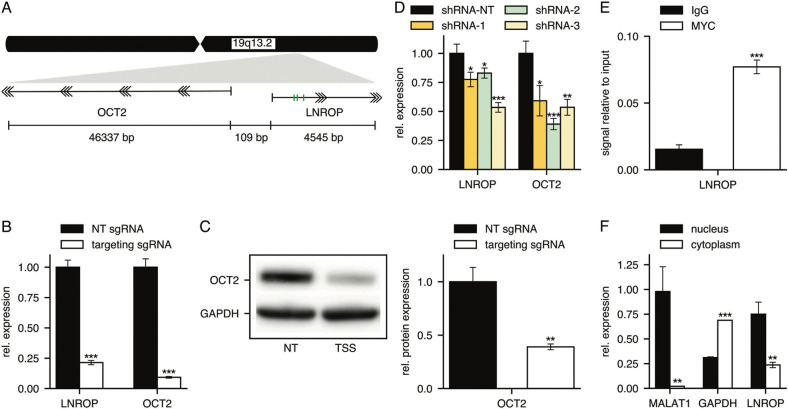
Functional characterization of LNROP as a regulator of OCT2 expression in RAMOS cells. **A** Schematic representation of OCT2 and LNROP *loci* based on data retrieved from Genome Browser (Gencode v39). Green bars indicate the three canonical (5’-CACGTG-3’) MYC E-boxes present in the sequence of LNROP and located 1082, 1096 and 1334 base pairs away from the TSS. **B** RT-qPCR analysis for the expression of LNROP and OCT2 in RAMOS cells after CRISPRi using sgRNAs directed against the TSS of LNROP compared to non-targeting (NT) guides. Data are mean ± SD of three replicates. **C** Western blot analysis of OCT2 expression in RAMOS cells after CRISPRi-mediated downregulation of LNROP using sgRNAs directed against the TSS of LNROP and a non-targeting guide (NT) as control. Western blot image (*Left*) was analyzed using Fiji software to quantify the expression of OCT2 relative to the loading control GAPDH (*Right*). Data are mean ± SD of three replicates. **D** RT-qPCR analysis for the expression of LNROP and OCT2 in RAMOS cells after RNA interference using small-hairpin RNA constructs targeting 3 different sites of the LNROP sequence. Data are mean ± SD of three replicates. **E** ChIP-qPCR analysis of MYC binding to the LNROP promoter in RAMOS cells. Normal Rabbit IgG was used as control. Data are mean ± SD of three replicates. **F** RT-qPCR analysis for the cytoplasmic/nuclear distribution of LNROP transcripts after cellular fractionation. MALAT1 and GAPDH served as nuclear and cytoplasmic markers, respectively. Data are mean ± SD of three replicates. *P < 0.05; **P < 0.01; ***P < 0.001, unpaired *t* test.

In the genome, LNROP is antisense to POU2F2, the gene encoding the transcription factor octamer-binding protein 2 (OCT2). Both genes are separated by only 109 base pairs (Fig. 1A). LNROP is also the host gene of MIR4323, a micro-RNA of unknown function but of potential use as a biomarker [40–44]. OCT2 is part of the POU transcription factor family, which binds DNA through specific POU domains to regulate transcription [45–48]. OCT2 binds immunoglobulin gene promoters and plays a key role in the proliferation and differentiation of human B cells [45–51]. OCT2 expression is essential for the proliferation and survival of diffuse large B cell lymphoma [49].

Here we show that LNROP is predominantly present in the nucleus and a direct target of MYC. Using standard CRISPR-Cas9 gene inactivation and a library of sgRNAs, we identified key spots in the sequence of LNROP where the insertion of mutations and INDELs leads to downregulation of its expression and results in defects of cell proliferation. Our data suggest that LNROP functions as a cis-acting regulator of OCT2 and through that link affects multiple downstream targets. One of these targets is SHP-1 (PTPN6, protein tyrosine phosphatase non-receptor type 6), a tyrosine phosphatase with anti-proliferative activity in B cells. SHP-1 is negatively regulated by OCT2.

## Results

### Downregulation of LNROP using CRISPRi or shRNA results in reduced expression of OCT2

MYC controls the expression of numerous lncRNAs. Several of these lncRNAs are essential for maintaining the proliferation of human lymphoid cells. In a CRISPRi screen, the sgRNAs targeting the TSS of these lncRNAs are depleted from the library [30, 31]. One of the lncRNAs identified in this way as required for lymphoid cell proliferation is LNROP (ENSG00000254887). In the genome, the TSS of LNROP is 109 bp downstream of the TSS of OCT2 (POU2F2) (Fig. 1A). The proximity of LNROP and OCT2 transcription start sites raised the possibility that LNROP expression might influence the expression of OCT2. To address this question, we used targeted CRISPRi. Guide sequences targeting the TSS of LNROP were cloned into the lenti-guide(MS2)_puroT2AGFP vector containing the puromycin resistance gene. A non-targeting sequence was used as control. Burkitt lymphoma-derived RAMOS cells stably expressing dCas9 fused to the repressor domain SID [32] were transduced with such guides and kept in culture for a period of 7 days in the presence of puromycin before total RNA and protein extraction. The expression of both LNROP and OCT2 was reduced up to 75% relative to the control when analyzed by RT-qPCR (Fig. 1B). In the case of OCT2, protein levels were under 50% according to western blot quantification (Fig. 1C). LNROP and OCT2 are oriented in a head-to-head configuration and share a promoter region (Fig. 1A). LNROP-targeted CRISPRi could therefore interfere directly with the promoter of OCT2, or LNROP could independently regulate OCT2. To address this issue, we designed three different short hairpin RNAs (shRNAs) directed against the LNROP transcript. The shRNAs and a non-targeting sequence were cloned into the pLKO.1_puro vector, which also contained a puromycin resistance gene. RAMOS cells were transduced with the lentiviral preparation and cultured in the presence of puromycin to obtain a population that stably expresses shRNAs. Ten days after transduction, we obtained total RNA from the cells and measured the expression of LNROP and OCT2 by RT-qPCR (Fig. 1D). Although the downregulation of LNROP with this method was not as strong as the one achieved with CRISPRi, we observed a concomitant downregulation of OCT2 with either of the three shRNAs. This result supports the hypothesis that LNROP independently regulates OCT2 and that the CRISPRi results do not reflect an off-target activity of the sgRNA constructs.

### LNROP is a direct transcriptional target of MYC

LNROP was identified as a potential target of MYC, because its expression is highly sensitive to the level of MYC in P493-6 cells [30]. LNROP contains three canonical (5′-CACGTG-3′) MYC E-boxes adjacent to its TSS (Fig. 1A), and previous ChIPseq experiments in K562 and MCF-7 cell lines demonstrate that MYC can effectively bind to this promoter region [52]. To confirm MYC-dependent expression of LNROP in RAMOS cells, we performed a ChIP analysis followed by qPCR. Chromatin isolated by using an anti-MYC antibody showed a strong enrichment in the TSS of LNROP relative to the IgG control antibody in RAMOS cells (Fig. 1E). This enrichment, together with previous results [30, 52], indicates that MYC binds directly to the LNROP promoter region, and that LNROP is a direct transcriptional target of MYC.

### LNROP is abundantly present in the nucleus

Many human lncRNAs localize in the nucleus and function in the regulation of gene expression [10, 12, 13, 15–17]. We hypothesize that LNROP is acting as a key intermediary between MYC and OCT2. Total RNA isolated from nuclear and cytoplasmic fractions was analyzed by RT-qPCR to determine the relative abundance of LNROP in different subcellular compartments. LNROP is significantly enriched in the nuclear fraction, where up to 75% of all transcripts accumulate (Fig. 1F). To ensure that we achieved a correct cellular fractionation, we also analyzed the distribution of MALAT1 and GAPDH, whose transcripts serve as controls for the nuclear and cytoplasmic fractions, respectively [53, 54].

### Disruption of the LNROP sequence by standard CRISPR-Cas9 leads to downregulation of OCT2

Because CRISPRi on genes with shared promoters is inconclusive and shRNAs against LNROP were inefficient, we used standard CRISPR-Cas9 to disrupt the sequence and function of LNROP. LNROP is non-coding, and in general lncRNAs are not susceptible to inactivation through the INDELs produced by CRISPR-Cas9 double-strand breaks and subsequent repair. However, lncRNAs do have specific regions which are critical for function. To target these unknown critical regions, we designed a library of 451 sgRNAs targeting the sequence of LNROP. As a positive control for this technique, we included 2 459 guides targeting DNM2, a protein-coding gene that is essential for cell survival and proliferation [55, 56]. The designed guides were synthesized in microarray format and the resulting guide pool was amplified and cloned in library format into the lenti-guide(MS2)_puroT2AGFP vector containing a puromycin resistance gene.

**Fig. 2:**
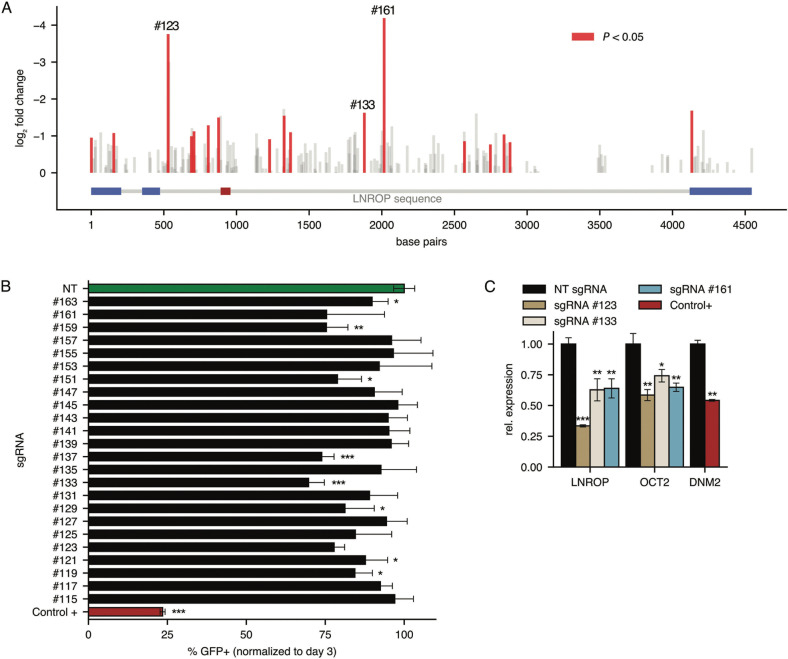
Phenotypic characterization and OCT2 expression analysis after targeting LNROP using CRISPR-Cas9. **A** Schematic representation of the sgRNAs library used to target LNROP in RAMOS cells stably expressing active Cas9. Bars correspond to individual guides, and they are represented according to their position over LNROP sequence where introns are colored in grey, exons in blue and MIR4323 in red. Bar height corresponds to the log_2_ fold change in guide abundance as measured by sequencing. Red bars indicate significantly depleted sgRNAs (P < 0.05). Guides (#123, #133 and #161) selected for subsequent experiments are labeled at their positions. **B** Phenotype characterization of RAMOS after standard CRISPR-Cas9 using sgRNAs targeting the LNROP sequence and expressed along with GFP. The number of GFP-expressing cells present in cultures was measured after 3 and 49 days using flow cytometry. Results indicate the change in the presence of GFP-expressing cells, using day 3 as starting point. A non-targeting guide (green) and a guide targeting DNM2 (red) were used as negative and positive controls, respectively. Data are mean ± SD of three replicates. **C** RT-qPCR analysis of LNROP and OCT2 expression in RAMOS cells after standard CRISPR-Cas9 using three sgRNAs (#123, #133 and #161) targeting the LNROP sequence or a non-targeting guide (NT). Data are mean ± SD of three replicates. *P < 0.05; **P < 0.01; ***P < 0.001, unpaired *t* test.

Cas9-expressing and wild-type RAMOS cells were transduced with the lentiviral pool carrying the library and selected with puromycin for two days. After selection, the cells were kept in culture for 14 days. At that time, guide sequences were PCR-amplified from genomic DNA, and the amplicons were subjected to next-generation sequencing [57] to measure the relative abundance of guides. Guides that mediate a disruption of gene functions essential for cell proliferation are depleted under these experimental conditions. For LNROP, several guides were highly diminished or missing (Fig. 2A). As expected, numerous guides targeting DNM2 exons or introns were also depleted. The guide targeting DNM2 that was more efficiently depleted from the library (log_2_ fold change in guide abundance of -8.51) was used as positive control in subsequent experiments.

We then validated these findings by a different method. We selected several significantly depleted LNROP and DNM2 guides, avoiding overlapping guides and guides targeting MIR4323, and determined the effect of these guides on cell proliferation using GFP as a read-out [32, 58]. We used these guides in a competition assay performed with GFP-expressing sgRNA constructs [32, 58]. Individual guides were transduced into Cas9-expressing RAMOS cells at low multiplicity of infection, including positive control guides targeting DNM2 and non-targeting guides. Cells were kept in culture for seven weeks without puromycin selection. GFP served as an indicator for the fraction of transduced cells that survived. Guides targeting LNROP induced a decrease in the fraction of GFP-expressing cells as did guides targeting DNM2 (Fig. 2B). Non-targeting guides had no detectable effect on the fraction of GFP-expressing cells.

In a different approach, the GFP-expressing sgRNA constructs were again transduced into Cas9-expressing RAMOS cells, and GFP-expressing cells were isolated three days after transduction via flow cytometry. Total RNA was isolated from sorted cells and used to perform RT-qPCR. Cells transduced with non-targeting guides served as control to normalize expression levels. The guides targeting LNROP caused a reduction in the expression of both LNROP and OCT2 (Fig. 2C). These results further document the link between LNROP and OCT2 expression and illustrate the importance of LNROP in cell proliferation.

### Overexpression of LNROP in situ induces an increase in OCT2 but ectopic overexpression of LNROP has no effect on OCT2 levels

**Fig. 3:**
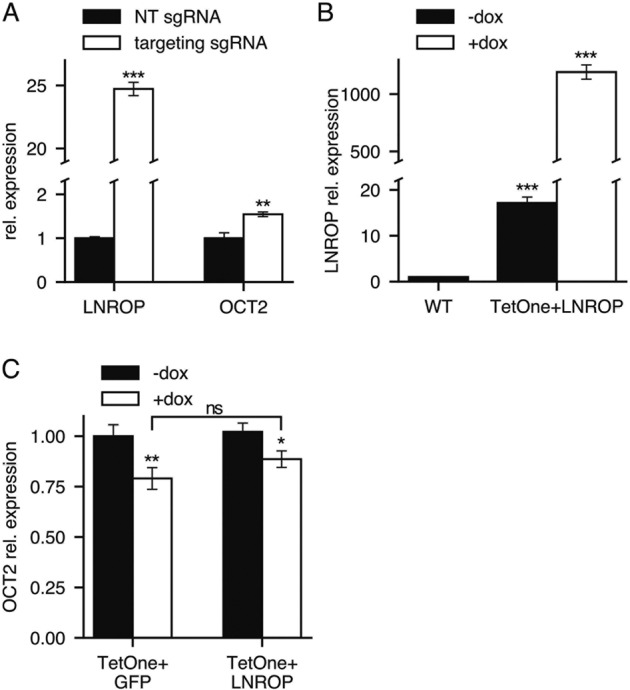
Analysis of LNROP and OCT2 expression in RAMOS cells after LNROP overexpression using CRISPRa or ectopic copy transduction. **A** RT-qPCR analysis of LNROP and OCT2 expression in RAMOS cells after CRISPR activation using a non-targeting guide (NT) or a targeting sgRNA directed against the TSS of LNROP. Data are mean ± SD of three replicates. **B** RT-qPCR analysis of LNROP expression in RAMOS cells that are wild type (WT) or transduced with a doxycycline-inducible ectopic copy of LNROP. Expression of LNROP was measured 48 hours after doxycycline addition. Data are mean ± SD of three replicates. **C** RT-qPCR analysis of OCT2 expression in RAMOS cells transduced with a doxycycline-inducible ectopic copy of GFP (control) or LNROP. Expression of OCT2 was measured 48 hours after doxycycline addition. Data are mean ± SD of three replicates. *P < 0.05; **P < 0.01; ***P < 0.001; ns, not significant, unpaired *t* test.

As a further investigation of the link between LNROP and OCT2, we overexpressed LNROP using two different experimental approaches. In the first, we upregulated LNROP expression through CRISPRa. The same sgRNAs and lentiviral vectors used in CRISPRi were transduced into RAMOS cells that stably expressed the synergistic activation mediator (dCas9-SAM) [59]. After transduction, the cells were cultured for 7 days in the presence of puromycin followed by extraction of total RNA. The relative expression of LNROP and OCT2 was measured by RT-qPCR and compared to the expression in cells transduced with non-targeting guides. Guides targeting the TSS of LNROP induced an overexpression of the gene by up to 25 times as compared to negative controls. This hyperactivation of the long non-coding transcription was accompanied by a moderate increase in OCT2 expression of up to 1.5-fold (Fig. 3A). As mentioned before, we cannot rule out the possibility that we are directly interfering with the TSS of OCT2, so we decided to try a second approach to achieve LNROP overexpression. We retrotranscribed its sequence from total RNA and cloned it into the tetracycline-inducible gene expression system, TetOne, along with the puromycin resistance gene. RAMOS cells were transduced with this TetOne-containing lentivirus preparation and cultured in Tet-free media and in the presence of puromycin for 7 days. Following recovery, doxycycline was added to induce the ectopic expression of LNROP. Two days after induction, RNA was extracted and the levels of LNROP were determined by RT-qPCR. A version of the TetOne plasmid containing the coding sequence of GFP instead of LNROP served as negative control. This ectopic expression of LNROP achieved levels of more than 1 000 times over wildtype cells (Fig. 3B). Yet, this flood of LNROP did not induce a significant increase in the expression of OCT2 (Fig. 3C). We noticed a small reduction in OCT2 levels in the presence of doxycycline. However, a similar reduction was observed in cells expressing an ectopic copy of GFP (Fig. 3C), probably due to the exposure to the doxycycline itself. These results suggest that LNROP acts in cis to regulate the expression of OCT2.

### The interaction between LNROP and OCT2 is unidirectional

**Fig. 4:**
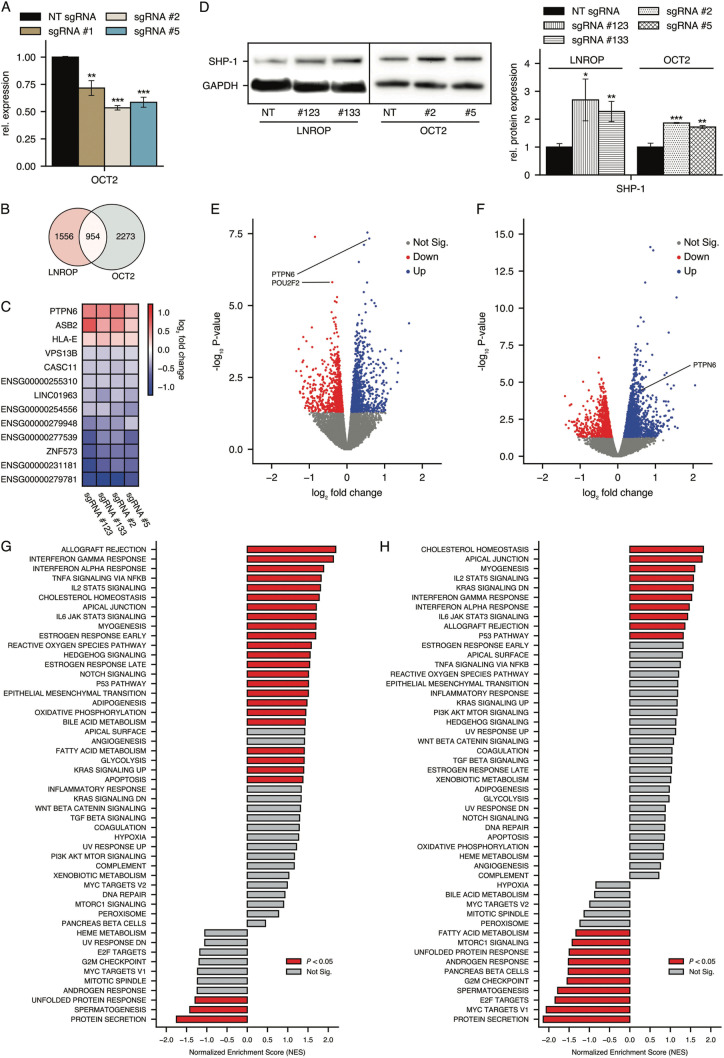
Characterization of the effects of CRISPR-Cas9 mediated disruption of LNROP and OCT2 on gene expression and downstream pathways in RAMOS cells. **A** RT-qPCR analysis of OCT2 expression in RAMOS cells after standard CRISPR-Cas9 mediated inactivation using three sgRNAs (#1, #2 and #5) targeting OCT2 exons or a non-targeting guide (NT). Data are mean ± SD of three replicates. **B** Venn diagram illustrating the pool of all genes with significantly altered expression after the disruption of either LNROP or OCT2 by standard CRISPR-Cas9 and at least one guide per gene (#123 or #133 for LNROP; #2 or #5 for OCT2), based on RNAseq data. **C** Heatmap showing log_2_ fold change for genes with altered expression that showed up in sequencing data in all experimental conditions (guides #123 and #133 for LNROP and guides #2 and #5 for OCT2). **D** Western blot analysis of SHP-1 expression in RAMOS cells after CRISPR-mediated downregulation of LNROP using targeting guides #123 or #133, OCT2 using targeting guides #2 or #5 and a non-targeting guide (NT) as control. Western blot image (*Left*) was analyzed using Fiji software to quantify the expression of SHP-1 relative to the loading control GAPDH (*Right*). Data are mean ± SD of two replicates. **E, F** Volcano plots showing the log_2_ fold change for all genes measured by RNAseq after disruption of LNROP (**E**) or OCT2 (**F**) sequence by standard CRISPR-Cas9 and using guides #123 and #2, respectively. Genes with significant (*P* < 0.05) changes in expression are colored in red (up-regulated) or blue (down-regulated). **G, H** GSEA Hallmark analysis of all genes with altered expression after disruption of LNROP (**G**) or OCT2 (H) sequence by standard CRISPR-Cas9 and using guides #123 and #2, respectively. Pathways that are significantly enriched are colored in red (*P* < 0.05). A positive Normalized Enrichment Score (NES) value indicates enrichment upon LNROP or OCT2 downregulation, a negative NES indicates enrichment in the control. *P < 0.05; **P < 0.01; ***P < 0.001, unpaired *t* test.

OCT2 is a transcription factor and regulates multiple genes. This suggests that changes in LNROP and OCT2 could cause alterations in the cellular transcriptome and could reveal information on the downstream effects of the OCT2-LNROP axis. To explore this possibility, we downregulated LNROP and OCT2 separately using the standard CRISPR-Cas9 approach. For LNROP, we used guide RNAs #123 and #133 identified as effective in the experiments described above. For OCT2, we designed guides targeting the exon region of the gene and identified three effective sgRNAs (Fig. 4A). We then transduced cells with LNROP guides #123 and #133 and, in separate cultures, with OCT2 guides #2 and #5. Non-targeting guides were used as controls. Total RNA extracted from cells was subjected to ribosomal RNA removal followed by next-generation sequencing.

Disruption of LNROP and of OCT2 produced two groups of genes with significantly altered expression (Fig. 4E and F). A gene set enrichment analysis (GSEA) of normalized counts for these genes using the Hallmark database showed that previously identified pathways controlled by OCT2, such as the JAK/STAT3 signaling axis, type I IFN response or genes regulated by NF-*κ*B [49], are disrupted under LNROP downregulation (Fig. 4G). Additionally, a comparison of these two lists showed that whereas the disruption of LNROP downregulates OCT2, the reverse is not the case. Therefore, the path of interactions from LNROP to OCT2 is unidirectional. LNROP affects OCT2, but OCT2 does not affect LNROP.

### Downregulation of LNROP and of OCT2 is followed by upregulation of SHP-1

We also identified several genes that showed significant changes in the same direction by the disruption of LNROP or OCT2 (Fig. 4B and C; Table 1). We used one of the shared target genes, SHP-1, to illustrate the downstream reach of LNROP. SHP-1 is significantly upregulated at the RNA and protein levels by the disruption of LNROP and of OCT2 (Fig. 4D). SHP-1 plays important roles in cell proliferation, differentiation, and oncogenic transformation [60–65]. In B cells, SHP-1 has an anti-proliferative effect attenuating pathways initiated by growth factors, cytokines and the immunoreceptor tyrosine-based activation motive (ITAM)-containing receptors [66]. Furthermore, the GSEA analysis of genes with altered expression upon LNROP disruption showed several pathways that are regulated by SHP-1 (STAT, IFN, TNFR) [60, 66, 67] (Fig. 4G). These results suggest that SHP-1 is among the pool of genes that are downstream of the LNROP-OCT2 axis and are implicated in MYC-driven B cell proliferation.

**Table 1:** Genes with altered expression that showed up in sequencing data from both guides against LNROP and OCT2.

Gene	logFC	*P v*alue
PTPN6	0.590004	0.000000
ASB2	0.531171	0.000002
CASC11	−0.295422	0.000054
ENSG00000254556	−0.478493	0.000139
HLA-E	0.219195	0.000200
ENSG00000279948	−0.444941	0.000359
ENSG00000255310	−0.270265	0.000719
LINC01963	−0.377894	0.000813
VPS13B	−0.208463	0.000929
ENSG00000277539	−0.690732	0.002482
ZNF573	−0.611349	0.003620
ENSG00000279781	−1.168887	0.006780
ENSG00000231181	−0.665388	0.021015

This means that these genes presented altered expression in all experimental conditions (guides #123 and #133 for LNROP and guides #2 and #5 for OCT2). Ordered by PValue. Only showing logFC and *P v*alue for the most efficient guides.

## Discussion

There is a category of lncRNAs that are both regulated by MYC and required to sustain MYC-driven cell proliferation. Examples of these long non-coding transcripts are SNHG17 [37], SNAI3-AS1 [68], MIR17HG [69], SNHG26 [38], SNHG5 [70, 71], and numerous others [32]. All of these also exert a pro-growth effect on the cell. They can be collectively classified as “MYC enablers”. This class of lncRNAs is likely to play a significant role in the oncogenicity of MYC.

LNROP has all the attributes of a MYC enabler. It is regulated by MYC and is required for MYC-dependent proliferation of human B cells. LNROP targets POU2F2, the gene encoding the transcription factor OCT2. LNROP and POU2F2 occupy adjacent positions in the genome, their TSS are only 109 bases apart, and their transcription proceeds in opposite directions. The transcript of LNROP is predominantly located in the cell nucleus. Downregulation of LNROP by shRNA, CRISPRi or standard CRISPR-Cas9 results in reduced expression of POU2F2 and OCT2, whereas upregulation of LNROP by CRISPRa is reflected in enhanced expression of POU2F2. However, ectopic overexpression of LNROP has no effect on the expression of POU2F2. These observations suggest that LNROP is a cis-acting regulator of POU2F2-OCT2.

The link between LNROP and OCT2 is unidirectional as mutational inactivation of OCT2 does not change the expression of LNROP. When LNROP and OCT2 are downregulated by CRISPR-Cas9 in separate cell cultures, both cultures show significant changes in the transcriptome. Some differentially regulated genes are identical in both cultures and may represent the effect of LNROP on the transcriptional regulatory activity of OCT2.

OCT2 is a transcription factor with numerous target genes that perform essential functions in the development and differentiation of B cells and in the growth of hematopoietic malignancies [49–51] and solid tumors [49].

Relevant pathways are significantly disrupted during the independent downregulations of LNROP and OCT2 (Fig. 4G and H), and we can observe previously known targets of OCT2, such as the IL-6/JAK/STAT3 signaling axis or the genes that respond to type I IFN [49].

From the differentially expressed genes identified by OCT2 CRISPR-Cas9, we selected SHP-1 to exemplify the downstream effects of the LNROP-OCT2 interaction. SHP-1 is a tyrosine phosphatase that exerts significant inhibitory activities on programs stimulated by OCT2 [72–74] and is negatively regulated by OCT2. It is upregulated when OCT2 is inactivated by CRISPR-Cas-9, showing that its basal activity of SHP-1 in B cells is controlled by OCT2.

**Fig. 5:**
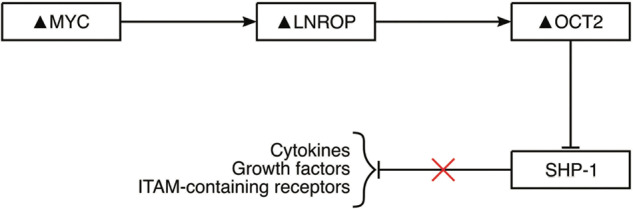
Schematic representation of the lines of interaction studied in this article. The MYC-regulated lncRNA LNROP promotes the expression of the transcription factor OCT2 and acts as a key intermediary between MYC and OCT2. SHP-1, a transcription factor with anti-proliferative activity in B cells, is downregulated by OCT2.

The lines of interaction studied in this report can be summarized as follows: LNROP functions as an essential component of MYC-stimulated B cell proliferation. It is a positive regulator of OCT2 which in turn is a positive regulator of B cell development and differentiation. Among the numerous targets of OCT2, we present as an example SHP-1 which interferes with normal B cell development and is negatively regulated by OCT2 (Fig. 5).

Our studies raise the question as to the mechanism of the LNROP-OCT2 interaction. LNROP could function as a recruiter of transcription factor and enhancer elements for OCT2. It could serve as a scaffold for diverse stimulators of transcription, or it could interfere with negative regulation of OCT2 transcription. The possibility that MIR4323 plays a part in the LNROP-OCT2 interaction needs to be examined, beginning with a molecular characterization of the functions of this microRNA. For a complete understanding of the LNROP-OCT2 axis, additional studies will be required.

## Materials/subjects and methods

### Cell cultures and transduction

RAMOS cells (National Cancer Institute, Bethesda, MD, USA) were cultured in suspension in Gibco RPMI 1640 media (Thermo Fisher Scientific, Hanover Park, IL, USA) and split when necessary to keep cell density between 0.1 and 2 million cells/mL. 293 T cells were cultured in Gibco DMEM (Thermo Fisher Scientific), and passages were made to keep cultures below 90% confluence. In both cases, we obtained complete culture media by supplementing with 10% (vol/vol) Fetal Bovine Serum (FBS, Omega Scientific, Tarzana, CA, USA) and 100 U/mL penicillin, 0.1 mg/mL streptomycin, 2 mM glutamine (Thermo Fisher Scientific). FBS was substituted by tetracycline-free FBS (Omega Scientific) for cells transduced with plasmids carrying a tetracycline-inducible promoter. Cultures were routinely maintained in an incubator at 95% RH, 5% CO_2_ and 37 °C.

Lentivirus particles were produced by transfecting 293 T cells with the appropriate constructs along with the plasmids required for packaging (Addgene, Watertown, MA, USA; pRSV-Rev, 12253; pMDLg/pRRE, 12251; and pMD2.G, 12259) [75]. Lipofectamine 3000 (Life Technologies, Grand Island, NY, USA) was used for transfection following manufacturer’s instructions. Lentivirus-containing cell culture media was collected 48 hours after transfection and stored at -80 °C before use without any further purification. RAMOS cells were transduced using lentivirus preparations, and, after 48 hours, they were selected with 7.5 µg/mL blasticidin (Thermo Fisher Scientific) or 1 µg/mL puromycin (Sigma-Aldrich, St. Louis, MO, USA), as appropriate.

### DNA constructs

shRNA sequences were synthesized by Integrated DNA Technologies (IDT, Coralville, IA, USA) and cloned into the AgeI/EcoRI sites of the pLKO.1_puro vector (Addgene; 8453) [76]. The reverse-transcribed sequence of the LNROP transcript or the GFP-coding sequence (control) were cloned into EcoRI/BamHI sites of the pLVX-TetOne_puro vector (Takara Bio, Mountain View, CA, USA; Clontech, 631849). Lenti-dCas9-SID_blast and lenti-guide(MS2)_puroT2AGFP were obtained as previously described [32]. All primers are listed in Table S1.

### Cellular fractionation

Cellular fractionation was performed as previously described by Roberts *et al*. [77]. Briefly, cells were collected by centrifugation at 300 g and washed twice with PBS. Cell pellets were then suspended in a lysis buffer containing 10 mM Tris-HCl pH 7.4, 10 mM NaCl, 3 mM MgCl_2_, and 0.5% IGEPAL, and incubated in ice for 5 minutes. Nuclei were then separated by centrifugation at 300 g for 5 min, and the supernatant which contained the cytoplasmic fraction was removed. Nuclei were washed twice in lysis buffer before RNA extraction. Both fractions were used to isolate total RNA using a Maxwell RSC simplyRNA Cells Kit (Promega, Madison, WI, USA) following the protocol provided by the manufacturer.

### RT-qPCR

Complementary DNA (cDNA) was obtained by reverse transcription (RT) of total RNA through random priming using the High-Capacity cDNA Synthesis Kit (Thermo Fisher Scientific) according to manufacturer’s instructions. Quantitative PCR (qPCR) was performed in a LightCycler 96 real-time PCR instrument (Roche, Indianapolis, IN, USA) using the Power SYBR Green PCR Master Mix (Thermo Fisher Scientific) and the previously synthesized cDNA as template. We used universal cycling conditions (95 °C for 10 minutes followed by 45 cycles of 95 °C for 10 s, 60 °C for 10 s, and 72 °C for 10 s) and the specificity of the reaction was confirmed by melting curve analysis. The standard curve method for relative quantification was used to obtain relative expression values. The complete list of primers can be found in Table S1.

### Chromatin immunoprecipitation (ChIP)

ChIP was performed using the SimpleChIP^®^ Enzymatic Chromatin IP Kit (Cell Signaling Technology, Danvers, MA, USA; #9003). A total of 1×10^7^ RAMOS wildtype cells per sample were used. Cell pellets were collected by centrifugation at 300 g for 10 minutes, washed twice in ice-cold PBS, resuspended in PBS containing 1% formaldehyde and incubated for 10 minutes before glycine neutralization. Cross-linked cells were washed twice in ice-cold PBS and resuspended in kit buffers for the isolation of nuclei. Chromatin fragmentation was performed enzymatically by adding 0.5 µL of Micrococcal Nuclease (Cell Signaling Technology; #10011) and incubation at 37 °C for 20 minutes to obtain the appropriate length of DNA fragments. ChIP was performed according to manufacturer’s instructions and using 10 µg of digested, cross-linked chromatin and anti-MYC antibody (Cell Signaling Technology; #9402). Histone H3 (D2B12) XP^®^ Rabbit mAb (Cell Signaling Technology; #4620) and Normal Rabbit IgG (Cell Signaling Technology; #2729) were used as positive and negative controls, respectively. Incubation in the presence of antibodies was performed overnight at 4 °C with rotation. Before incubation, 10 µL were set aside to use as 2% input sample. Chromatin elution and DNA purification were made using buffers provided with the kit. Samples were analyzed by qPCR using 2 µL of purified DNA per reaction and the primers listed in Table S1. The following formula was used to calculate signal relative to input: Percent input = 2% × 2^(C[T] 2% Input Sample – C[T] IP Sample)^; C[T] = CT = threshold cycle of PCR.

### CRISPR-Cas9 growth competition assays

Proliferative defects in cells were measured using a CRISPR-Cas9-based growth competition assays following published protocols [58]. The purpose of this experiment was to confirm that guides targeting the lncRNA have a negative impact over proliferation. To that end, we expressed each guide along with a coding sequence of GFP. This procedure allows monitoring the percentage of the population that is expressing the sgRNA/GFP using flow cytometry. Cells expressing sgRNAs that cause a defect in proliferation show a lower growth rate than non-transfected cells, and therefore the percentage of GFP-expressing cells decreases over time. Selected sgRNAs were individually synthesized by IDT and cloned into the lenti-guide(MS2)_PuroT2AGFP vector. The resulting construction was transduced in 293 T cells along with the necessary packaging plasmids to generate lentivirus particles. Constructs containing a non-targeting guide or a sgRNA targeting DNM2 sequence were used as negative and positive controls, respectively. RAMOS cells, at a density of 0.1 million cells per mL and stably expressing active Cas9, were transduced with the lentivirus preparation in 24-well plates. The efficiency of transduction was 10-15%, and cultures were split every two days to keep cell density below 2 million cells per mL. The percentage of GFP-expressing cells was measured by flow cytometry in a Novocyte instrument (ACEA Biosciences, San Diego, CA, USA) after 3 and 49 days. Data were processed in FlowJo™ v10 Software (BD Biosciences, San Jose, CA, USA).

### Standard CRISPR sgRNA library preparation

The genomic sequence of LNROP was retrieved from Gencode v25 (https://www.gencodegenes.org/human/release_25.html, accessed 11 December 2016) [78] and guides were designed to cover the whole locus, including introns. We obtained a total of 451 guides after excluding those targeting highly repetitive sequences, the microRNA MIR4323 or guides with exact matches in other parts of the genome. Additionally, 2 459 guides targeting the coding gene DNM2 were included as positive control following the same design criteria.

Guides were purchased from CustomArray and designed with flanking cloning sites. The library was first PCR-amplified and subsequently cloned into the lenti-sgRNA(MS2)_puro vector using the Gibson assembly kit (New England Biolabs, Ipswich, MA, USA; E5510S). Endura™ Electrocompetent Cells (Lucigen, Middleton, WI, USA; 60242) were transformed using the ligation product, and colonies (∼100× library coverage) were pooled into a liquid culture for plasmid isolation using QIAfilter Plasmid Midi Kit (Qiagen, Germantown, MA, USA; 12243).

### Standard CRISPR-Cas9 screening

The library containing sgRNAs targeting LNROP and DNM2 was used to prepare a lentivirus suspension as described in previous sections. Cas9-expressing and WT (library control) RAMOS cells were transduced with the lentivirus preparation and cultured for 48 hours. An estimated transduction efficiency of 10-15% was achieved. Transduced cells were selected in the presence of puromycin for 72 hours and recovered for 14 days. Cells were split when needed while maintaining at least 2.9 ×10^6^ cells to assure a ~1 000x library representation. Three replicates per cell line were transduced. Genomic DNA was extracted from 3×10^6^ cells per replica using the DNeasy Blood and Tissue Kit (Qiagen; 69504).

Genomic DNA was used as template for PCR amplification of guide sequences using primers with Illumina (San Diego, CA, USA) adaptors, sequencing primer binding sites and barcodes (Table S2). The Q5 Hot Start High-Fidelity 2X Master Mix (New England Biolabs; M0494) was used for amplification, with a total of 23 PCR cycles at an annealing temperature of 61 °C. The resulting amplicons were subjected to separation by electrophoresis in a 1.5% (wt/vol) TAE agarose gel and recovered using the QIAquick Gel Extraction Kit (Qiagen; 28704).

Barcode multiplexed samples were pooled and sequenced using AmpliconSeq on an Illumina NextSeq 500 instrument together with unrelated sequencing samples to obtain sequence diversity. Read lengths were 1 × 75 bp, and a read depth of 20 million per multiplexed library was used. The raw FASTQ file was divided into libraries using barcode sequences and FASTX (FASTX-Toolkit, RRID:SCR_005534, http://hannonlab.cshl.edu/fastx_toolkit/, accessed 6 January 2022). FASTQ files were processed using count_spacers.py [57] and designed guide sequences. Fold changes were determined using EdgeR (v3.36.0) [79].

### Western blotting

Cells were separated from media by centrifugation at 300 g for 10 minutes. Cell pellets were washed twice in ice-cold PBS and finally resuspended in RIPA buffer with protease inhibitors. Lysates were sonicated in a Qsonica q700 sonicator at 70% amplitude for 10 minutes for total nuclei disruption. Non-soluble cell parts were removed by centrifugation at 12 000 r.p.m. for 20 minutes and 4 °C. A total of 20 µg of protein per sample was completed with Bolt™ LDS Sample Buffer (Life Technologies; B0007) and Sample Reducing Agent (Life Technologies; B0009), boiled for 5 minutes and cooled in ice for at least 1 minute, before they were loaded into polyacrylamide gels. Samples were transferred to a PVDF membrane, blocked with 5% (wt/vol) milk and incubated overnight at 4 °C with antibodies for OCT2 (Invitrogen; #39-5400), SHP-1 (Cell Signaling Technology; #3759) or GAPDH (Cell Signaling Technology; #2118). After incubation with the appropriate secondary antibody, blots were developed in a ChemiDoc MP Imaging System. Relative protein amount estimation was made using Fiji software [80].

### Statistical analysis

Statistic analysis was performed using GraphPad Prism 8 software. All data are expressed as the means ± standard deviation (SD) for *n* independent experiments (indicated in figure legends). The two-tailed Student’s unpaired t test was used to analyze differences between two groups. Groups that were compared had a similar variance. Significance is indicated in the figures, and a *P* value <0.05 was considered statistically significant.

## Supplementary information

Supplemental information

Original western blot

Reproducibility checklist

## Data Availability

The datasets used and/or analyzed during the current study are available from the corresponding authors on reasonable request.

## References

[1] Eisenman RN (2001). Deconstructing myc. Genes Dev.

[2] Dang CV (2012). MYC on the path to cancer. Cell.

[3] Conacci-Sorrell M, McFerrin L, Eisenman RN (2014). An overview of MYC and its interactome. Cold Spring Harb Perspect Med.

[4] Blackwood EM, Eisenman RN (1991). Max: a helix-loop-helix zipper protein that forms a sequence-specific DNA-binding complex with Myc. Science.

[5] Eilers M, Eisenman RN (2008). Myc’s broad reach. Genes Dev.

[6] Gao J, Aksoy BA, Dogrusoz U, Dresdner G, Gross B, Sumer SO (2013). Integrative analysis of complex cancer genomics and clinical profiles using the cBioPortal. Sci Signal.

[7] Cerami E, Gao J, Dogrusoz U, Gross BE, Sumer SO, Aksoy BA (2012). The cBio cancer genomics portal: an open platform for exploring multidimensional cancer genomics data. Cancer Disco.

[8] Dalla-Favera R, Bregni M, Erikson J, Patterson D, Gallo RC, Croce CM (1982). Human c-myc onc gene is located on the region of chromosome 8 that is translocated in Burkitt lymphoma cells. Proc Natl Acad Sci USA.

[9] Mattick JS (2018). The State of Long Non-Coding RNA Biology. Noncoding Rna.

[10] Deveson IW, Hardwick SA, Mercer TR, Mattick JS (2017). The Dimensions, Dynamics, and Relevance of the Mammalian Noncoding Transcriptome. Trends Genet.

[11] Smith MA, Mattick JS (2017). Structural and Functional Annotation of Long Noncoding RNAs. Methods Mol Biol.

[12] Statello L, Guo CJ, Chen LL, Huarte M (2021). Gene regulation by long non-coding RNAs and its biological functions. Nat Rev Mol Cell Biol.

[13] Slack FJ, Chinnaiyan AM (2019). The Role of Non-coding RNAs in Oncology. Cell.

[14] Iyer MK, Niknafs YS, Malik R, Singhal U, Sahu A, Hosono Y (2015). The landscape of long noncoding RNAs in the human transcriptome. Nat Genet.

[15] Mumbach MR, Granja JM, Flynn RA, Roake CM, Satpathy AT, Rubin AJ (2019). HiChIRP reveals RNA-associated chromosome conformation. Nat Methods.

[16] Isoda T, Moore AJ, He Z, Chandra V, Aida M, Denholtz M (2017). Non-coding Transcription Instructs Chromatin Folding and Compartmentalization to Dictate Enhancer-Promoter Communication and T Cell Fate. Cell.

[17] Rinn JL, Chang HY (2012). Genome regulation by long noncoding RNAs. Annu Rev Biochem.

[18] Fatica A, Bozzoni I (2014). Long non-coding RNAs: new players in cell differentiation and development. Nat Rev Genet.

[19] Arman K, Moroy T (2020). Crosstalk Between MYC and lncRNAs in Hematological Malignancies. Front Oncol.

[20] Benetatos L, Benetatou A, Vartholomatos G (2020). Long non-coding RNAs and MYC association in hematological malignancies. Ann Hematol.

[21] Fatma H, Siddique HR (2020). Role of long non-coding RNAs and MYC interaction in cancer metastasis: A possible target for therapeutic intervention. Toxicol Appl Pharm.

[22] Swier L, Dzikiewicz-Krawczyk A, Winkle M, van den Berg A, Kluiver J (2019). Intricate crosstalk between MYC and non-coding RNAs regulates hallmarks of cancer. Mol Oncol.

[23] Iaccarino I (2017). lncRNAs and MYC: An Intricate Relationship. Int J Mol Sci.

[24] Deng K, Guo X, Wang H, Xia J (2014). The lncRNA-MYC regulatory network in cancer. Tumour Biol.

[25] Kim T, Jeon YJ, Cui R, Lee JH, Peng Y, Kim SH (2015). Role of MYC-regulated long noncoding RNAs in cell cycle regulation and tumorigenesis. J Natl Cancer Inst.

[26] Loven J, Orlando DA, Sigova AA, Lin CY, Rahl PB, Burge CB (2012). Revisiting global gene expression analysis. Cell.

[27] Sabo A, Kress TR, Pelizzola M, de Pretis S, Gorski MM, Tesi A (2014). Selective transcriptional regulation by Myc in cellular growth control and lymphomagenesis. Nature.

[28] Lin CY, Loven J, Rahl PB, Paranal RM, Burge CB, Bradner JE (2012). Transcriptional amplification in tumor cells with elevated c-Myc. Cell.

[29] Nie Z, Hu G, Wei G, Cui K, Yamane A, Resch W (2012). c-Myc is a universal amplifier of expressed genes in lymphocytes and embryonic stem cells. Cell.

[30] Hart JR, Roberts TC, Weinberg MS, Morris KV, Vogt PK (2014). MYC regulates the non-coding transcriptome. Oncotarget.

[31] Winkle M, van den Berg A, Tayari M, Sietzema J, Terpstra M, Kortman G (2015). Long noncoding RNAs as a novel component of the Myc transcriptional network. FASEB J.

[32] Raffeiner P, Hart JR, Garcia-Caballero D, Bar-Peled L, Weinberg MS, Vogt PK (2020). An MXD1-derived repressor peptide identifies noncoding mediators of MYC-driven cell proliferation. Proc Natl Acad Sci USA.

[33] Wang M, Gu J, Zhang X, Yang J, Zhang X, Fang X (2021). Long Non-coding RNA DANCR in Cancer: Roles, Mechanisms, and Implications. Front Cell Dev Biol.

[34] Zhang Z, Shu L, Hu M, Zhou X, Yang F, Zhou XH (2021). Emerging role of lncRNA DANCR in progenitor cells: beyond cancer. Eur Rev Med Pharm Sci.

[35] Thin KZ, Liu X, Feng X, Raveendran S, Tu JC (2018). LncRNA-DANCR: A valuable cancer related long non-coding RNA for human cancers. Pathol Res Pr.

[36] Tan F, Chen J, Du Z, Zhao F, Liu Y, Zhang Q (2022). MIR17HG: A Cancerogenic Long-Noncoding RNA in Different Cancers. Curr Pharm Des.

[37] Ma L, Gao J, Zhang N, Wang J, Xu T, Lei T (2022). Long noncoding RNA SNHG17: a novel molecule in human cancers. Cancer Cell Int.

[38] Jiang Q, Wang Z, Qi Q, Li J, Xin Y, Qiu J (2022). lncRNA SNHG26 promoted the growth, metastasis, and cisplatin resistance of tongue squamous cell carcinoma through PGK1/Akt/mTOR signal pathway. Mol Ther Oncolytics.

[39] Hegre SA, Samdal H, Klima A, Stovner EB, Norsett KG, Liabakk NB (2021). Joint changes in RNA, RNA polymerase II, and promoter activity through the cell cycle identify non-coding RNAs involved in proliferation. Sci Rep..

[40] Chakraborty S, Nath D (2022). A Study on microRNAs Targeting the Genes Overexpressed in Lung Cancer and their Codon Usage Patterns. Mol Biotechnol.

[41] Xia C, Li Q, Cheng X, Wu T, Gao P (2021). miR-4323 targets hepatoma-derived growth factor (HDGF) to suppress colorectal cancer cell proliferation. Pathol Res Pr.

[42] Handa T, Kuroha M, Nagai H, Shimoyama Y, Naito T, Moroi R (2021). Liquid Biopsy for Colorectal Adenoma: Is the Exosomal miRNA Derived From Organoid a Potential Diagnostic Biomarker?. Clin Transl Gastroenterol.

[43] Asakage M, Usui Y, Nezu N, Shimizu H, Tsubota K, Yamakawa N (2020). Comprehensive miRNA Analysis Using Serum From Patients With Noninfectious Uveitis. Invest Ophthalmol Vis Sci.

[44] Okumura T, Shimada Y, Omura T, Hirano K, Nagata T, Tsukada K (2015). MicroRNA profiles to predict postoperative prognosis in patients with small cell carcinoma of the esophagus. Anticancer Res.

[45] Phillips K, Luisi B (2000). The virtuoso of versatility: POU proteins that flex to fit. J Mol Biol.

[46] Sauter P, Matthias P (1998). Coactivator OBF-1 makes selective contacts with both the POU-specific domain and the POU homeodomain and acts as a molecular clamp on DNA. Mol Cell Biol.

[47] Latchman DS (1996). The Oct-2 transcription factor. Int J Biochem Cell Biol.

[48] Staudt LM, Clerc RG, Singh H, LeBowitz JH, Sharp PA, Baltimore D (1988). Cloning of a lymphoid-specific cDNA encoding a protein binding the regulatory octamer DNA motif. Science.

[49] Hodson DJ, Shaffer AL, Xiao W, Wright GW, Schmitz R, Phelan JD (2016). Regulation of normal B-cell differentiation and malignant B-cell survival by OCT2. Proc Natl Acad Sci USA.

[50] Song S, Cao C, Choukrallah MA, Tang F, Christofori G, Kohler H (2021). OBF1 and Oct factors control the germinal center transcriptional program. Blood.

[51] Advani AS, Lim K, Gibson S, Shadman M, Jin T, Copelan E (2010). OCT-2 expression and OCT-2/BOB.1 co-expression predict prognosis in patients with newly diagnosed acute myeloid leukemia. Leuk Lymphoma.

[52] Wang J, Zhuang J, Iyer S, Lin XY, Greven MC, Kim BH (2013). Factorbook.org: a Wiki-based database for transcription factor-binding data generated by the ENCODE consortium. Nucleic Acids Res.

[53] Miyagawa R, Tano K, Mizuno R, Nakamura Y, Ijiri K, Rakwal R (2012). Identification of cis- and trans-acting factors involved in the localization of MALAT-1 noncoding RNA to nuclear speckles. RNA.

[54] Tristan C, Shahani N, Sedlak TW, Sawa A (2011). The diverse functions of GAPDH: views from different subcellular compartments. Cell Signal.

[55] Wang T, Birsoy K, Hughes NW, Krupczak KM, Post Y, Wei JJ (2015). Identification and characterization of essential genes in the human genome. Science.

[56] Trochet D, Bitoun M (2021). A review of Dynamin 2 involvement in cancers highlights a promising therapeutic target. J Exp Clin Cancer Res.

[57] Joung J, Konermann S, Gootenberg JS, Abudayyeh OO, Platt RJ, Brigham MD (2017). Genome-scale CRISPR-Cas9 knockout and transcriptional activation screening. Nat Protoc.

[58] Erb MA, Scott TG, Li BE, Xie H, Paulk J, Seo HS (2017). Transcription control by the ENL YEATS domain in acute leukaemia. Nature.

[59] Konermann S, Brigham MD, Trevino AE, Joung J, Abudayyeh OO, Barcena C (2015). Genome-scale transcriptional activation by an engineered CRISPR-Cas9 complex. Nature.

[60] Varone A, Spano D, Corda D (2020). Shp1 in Solid Cancers and Their Therapy. Front Oncol.

[61] Dempke WCM, Uciechowski P, Fenchel K, Chevassut T (2018). Targeting SHP-1, 2 and SHIP Pathways: A Novel Strategy for Cancer Treatment?. Oncology.

[62] Kawakami T, Xiao W, Yasudo H, Kawakami Y (2012). Regulation of proliferation, survival, differentiation, and activation by the Signaling Platform for SHP-1 phosphatase. Adv Biol Regul.

[63] Lopez-Ruiz P, Rodriguez-Ubreva J, Cariaga AE, Cortes MA, Colas B (2011). SHP-1 in cell-cycle regulation. Anticancer Agents Med Chem.

[64] Wu C, Sun M, Liu L, Zhou GW (2003). The function of the protein tyrosine phosphatase SHP-1 in cancer. Gene.

[65] Hasler P, Zouali M (2001). B cell receptor signaling and autoimmunity. FASEB J.

[66] Neel BG, Gu H, Pao L (2003). The ‘Shp’ing news: SH2 domain-containing tyrosine phosphatases in cell signaling. Trends Biochem Sci.

[67] An H, Hou J, Zhou J, Zhao W, Xu H, Zheng Y (2008). Phosphatase SHP-1 promotes TLR- and RIG-I-activated production of type I interferon by inhibiting the kinase IRAK1. Nat Immunol.

[68] Li Y, Guo D, Lu G, Mohiuddin Chowdhury ATM, Zhang D, Ren M (2020). LncRNA SNAI3-AS1 promotes PEG10-mediated proliferation and metastasis via decoying of miR-27a-3p and miR-34a-5p in hepatocellular carcinoma. Cell Death Dis.

[69] Yuan J, Tan L, Yin Z, Zhu W, Tao K, Wang G (2019). MIR17HG-miR-18a/19a axis, regulated by interferon regulatory factor-1, promotes gastric cancer metastasis via Wnt/beta-catenin signalling. Cell Death Dis.

[70] Safa A, Gholipour M, Dinger ME, Taheri M, Ghafouri-Fard S (2020). The critical roles of lncRNAs in the pathogenesis of melanoma. Exp Mol Pathol.

[71] Li YH, Hu YQ, Wang SC, Li Y, Chen DM (2020). LncRNA SNHG5: A new budding star in human cancers. Gene.

[72] Getahun A, Beavers NA, Larson SR, Shlomchik MJ, Cambier JC (2016). Continuous inhibitory signaling by both SHP-1 and SHIP-1 pathways is required to maintain unresponsiveness of anergic B cells. J Exp Med.

[73] Li YF, Xu S, Ou X, Lam KP (2014). Shp1 signalling is required to establish the long-lived bone marrow plasma cell pool. Nat Commun.

[74] Tamir I, Dal Porto JM, Cambier JC (2000). Cytoplasmic protein tyrosine phosphatases SHP-1 and SHP-2: regulators of B cell signal transduction. Curr Opin Immunol.

[75] Dull T, Zufferey R, Kelly M, Mandel RJ, Nguyen M, Trono D (1998). A third-generation lentivirus vector with a conditional packaging system. J Virol.

[76] Stewart SA, Dykxhoorn DM, Palliser D, Mizuno H, Yu EY, An DS (2003). Lentivirus-delivered stable gene silencing by RNAi in primary cells. RNA.

[77] Roberts TC, Hart JR, Kaikkonen MU, Weinberg MS, Vogt PK, Morris KV (2015). Quantification of nascent transcription by bromouridine immunocapture nuclear run-on RT-qPCR. Nat Protoc.

[78] Frankish A, Diekhans M, Ferreira AM, Johnson R, Jungreis I, Loveland J (2019). GENCODE reference annotation for the human and mouse genomes. Nucleic Acids Res.

[79] Robinson MD, McCarthy DJ, Smyth GK (2010). edgeR: a Bioconductor package for differential expression analysis of digital gene expression data. Bioinformatics.

[80] Schindelin J, Arganda-Carreras I, Frise E, Kaynig V, Longair M, Pietzsch T (2012). Fiji: an open-source platform for biological-image analysis. Nat Methods.

