# Supplementary material for: The MYC-regulated lncRNA LNROP (ENSG00000254887) enables MYC-driven cell proliferation by controlling the expression of OCT2
Source: Cell Death Dis. 2023 Feb 27;14(2):168. doi: 10.1038/s41419-023-05683-6 (PMC9971199; doi:10.1038/s41419-023-05683-6)
Supplement: Supplementary file 1 — Supplemental information [file 41419_2023_5683_MOESM1_ESM.pdf]

Supplementary Information

Table S1. Selected oligodeoxynucleotide sequences.

	Forward sequence (5' to 3')	Reverse sequence (5' to 3')
**CRISPR guide sequences (Figs. 1B, 2B, 3C, 4A-C)**		
Non-targeting (NT)	CACCGGCGATTATTGAGATCGGCGT	AAACACGCCGATCTCAATAATCGCC
LNROP (TSS)	CACCGGGCTCGGCCCCCAGAGAGGG	AAACCCCTCTCTGGGGGCCGAGCCC
LNROP-sgRNA#123	CACCGTTGCTTGATCTTCCCTGCCG	AAACCGGCAGGGAAGATCAAGCAAC
LNROP-sgRNA#133	CACCGATGGGAGCTAGGGAGCAGTG	AAACCACTGCTCCCTAGCTCCCATC
LNROP-sgRNA#161	CACCGCTCAAGGCAGGGGGAGATGG	AAACCCATCTCCCCCTGCCTTGAGC
OCT2-sgRNA#1	CACCGAGTGGCTCAACGATGCAGGT	AAACACCTGCATCGTTGAGCCACTC
OCT2-sgRNA#2	CACCGGACGGGCAGCCAGCTAGCTG	AAACCAGCTAGCTGGCTGCCCGTCC
OCT2-sgRNA#5	CACCGCAGCTACCTCAGCAAACCCA	AAACTGGGTTTGCTGAGGTAGCTGC
**shRNA sequences (Fig. 1C)**		
NT-shRNA	CCGGTCCTAAGGTTAAGTCGCCCTCTCGAGAGGGCGACTTAACCTTAGGATTTTTG	AATTCAAAAATCCTAAGGTTAAGTCGCCCTCTCGAGAGGGCGACTTAACCTTAGGA
LNROP-shRNA-1	CCGGCAAGTCTGGAACAGGGAATGGCTCGAGCCATTCCCTGTTCCAGACTTGTTTTTG	CCGGACATTTCCTCATGTCCCTCAGCTCGAGCTGAGGGACATGAGGAAATGTTTTTTG
LNROP-shRNA-2	CCGGACATTTCCTCATGTCCCTCAGCTCGAGCTGAGGGACATGAGGAAATGTTTTTTG	AATTCAAAAAACATTTCCTCATGTCCCTCAGCTCGAGCTGAGGGACATGAGGAAATGT
LNROP-shRNA-3	CCGGATCTGACCTCAAGGAGCTAACCTCGAGGTTAGCTCCTTGAGGTCAGATTTTTTG	AATTCAAAAAATCTGACCTCAAGGAGCTAACCTCGAGGTTAGCTCCTTGAGGTCAGAT
**ChIP-qPCR primers (Fig. 1D)**		
LNROP	GACGATGGTCACTCCCATATAC	GGAAGTATGCTTGGGCACATA
control	GTGGAGCTCAAGACCACGTC	CTGACTGGTCAAGGGTGTGG
**RT-qPCR primers (Figs. 1B-C, 1E, 2B, 3A-C, 4A)**		
LNROP	CCTTCTTGGAGGCGTGAAAT	CTGTGAGCTGCAGAGGTATTC
MALAT1	GGAGAACTTCAGAAGAGCTTG	CACCAATCCCAACCGTAACAG
GAPDH	AACGGGAAGCTTGTCATCAATGGAAA	GCATCAGCAGAGGGGGCAGAG
OCT2	CAGCCACCTGCTCAGTTC	TTTGCTGAGGTAGCTGGAATAG
RPLP0 (housekeeping gene)	GAAACTCTGCATTCTCGCTTC	GGTGTAATCCGTCTCCACAG
**Cloning into TetOne (Fig. 3A-B)**		

LNROP	GCGGAATTCGGATAAGCAGGCTGGAAGAGCCGACTCC	GCGGGATCCTTGGCACATAATAGCATTTATTGGATATTTATTAAGTGATAAATTACCAAG
GFP	GCGGAATTCACCATGAGCAAGGGCGAGGAGCTGTTC	GCGGGATCCTTACTTGTACAGCTCGTCCATGCC

Table S2. PCR-primers used to generate guide library for next generation sequencing.

**Primer name**	**Sequence (5' to 3')**
Fwd_1 (i5-barcode)	AATGATACGGCGACCACCGAGATCTACACTCTTTCCCTACACGACGCTCTTCCGATCTTAAGTAGAGATATCTTGTGGAAAGGACGAAACACCG
Fwd_2 (i5-barcode)	AATGATACGGCGACCACCGAGATCTACACTCTTTCCCTACACGACGCTCTTCCGATCTTACACGATCATATCTTGTGGAAAGGACGAAACACCG
Fwd_3 (i5-barcode)	AATGATACGGCGACCACCGAGATCTACACTCTTTCCCTACACGACGCTCTTCCGATCTTAACGCATTATATCTTGTGGAAAGGACGAAACACCG
Fwd_4 (i5-barcode)	AATGATACGGCGACCACCGAGATCTACACTCTTTCCCTACACGACGCTCTTCCGATCTTCATGATCGATATCTTGTGGAAAGGACGAAACACCG
Fwd_5 (i5-barcode)	AATGATACGGCGACCACCGAGATCTACACTCTTTCCCTACACGACGCTCTTCCGATCTTCGTTACCAATATCTTGTGGAAAGGACGAAACACCG
Fwd_6 (i5-barcode)	AATGATACGGCGACCACCGAGATCTACACTCTTTCCCTACACGACGCTCTTCCGATCTTTCCTTGGTATATCTTGTGGAAAGGACGAAACACCG
Rev_1 (i7-barcode)	CAAGCAGAAGACGGCATACGAGATACAGGTATGTGACTGGAGTTCAGACGTGTGCTCTTCCGATCTGCCAAGTTGATAACGGACTAGCCTT
Rev_2 (i7-barcode)	CAAGCAGAAGACGGCATACGAGATAACGCATTGTGACTGGAGTTCAGACGTGTGCTCTTCCGATCTGCCAAGTTGATAACGGACTAGCCTT
