# Supplementary material for: The MYC-regulated lncRNA LNROP (ENSG00000254887) enables MYC-driven cell proliferation by controlling the expression of OCT2
Source: Cell Death Dis. 2023 Feb 27;14(2):168. doi: 10.1038/s41419-023-05683-6 (PMC9971199; doi:10.1038/s41419-023-05683-6)

Original western blots used in manuscript.

Fig. 1C: OCT2 (LNROP downregulation)

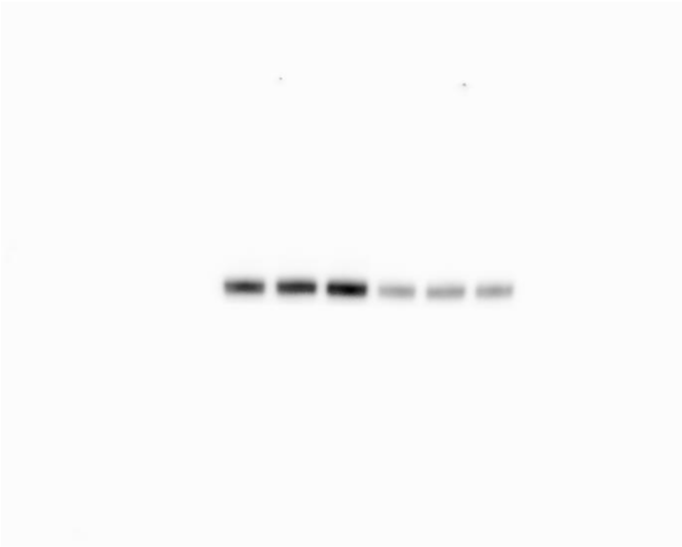

Fig. 1C: GAPDH (LNROP downregulation)

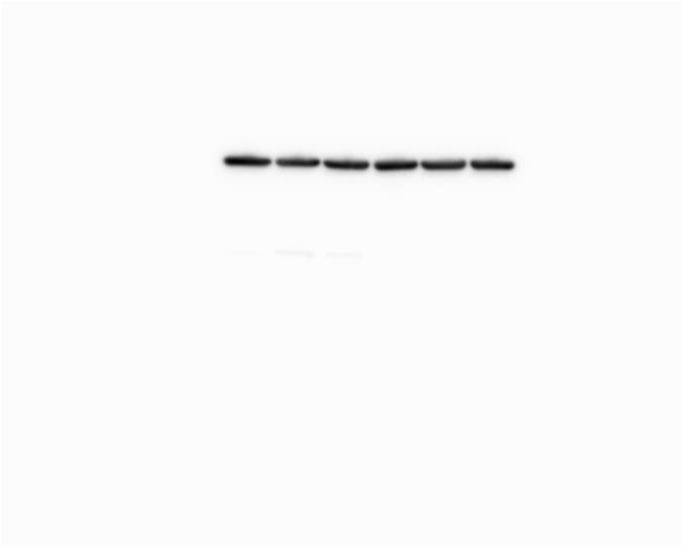

Fig. 4C (left): SHP-1 (LNROP downregulation)

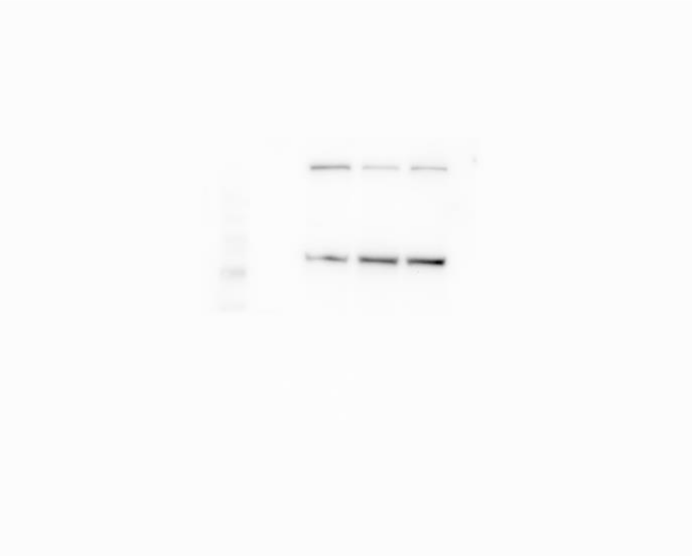

Fig. 4C (left): GAPDH (LNROP downregulation)

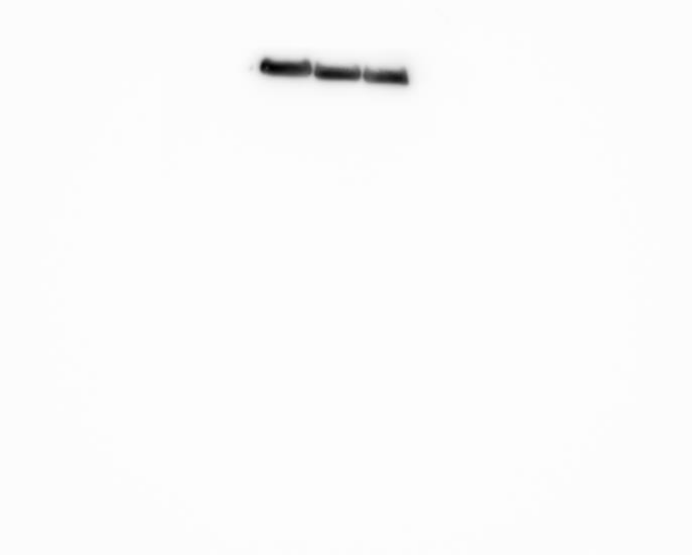

Fig. 4C (right): SHP-1 (OCT2 downregulation)

Fig. 4C (right): GAPDH (OCT2 downregulation)

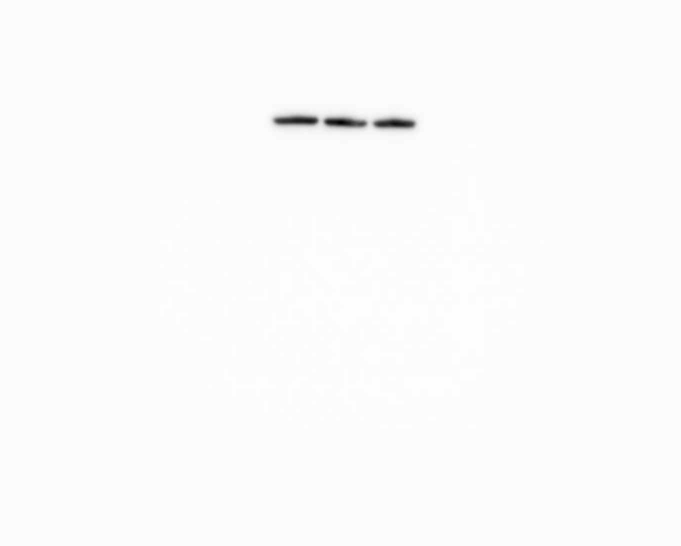

Supplement: Supplementary file 2 — Original western blot [file 41419_2023_5683_MOESM2_ESM.pdf]
